# Demonstrating the reliability of transdiagnostic mHealth Routine Outcome Monitoring in mental health services using experience sampling technology

**DOI:** 10.1371/journal.pone.0186294

**Published:** 2017-10-12

**Authors:** Simone J. W. Verhagen, Juliënne A. Berben, Carsten Leue, Anne Marsman, Philippe A. E. G. Delespaul, Jim van Os, Richel Lousberg

**Affiliations:** 1 Department of Psychiatry and Psychology, Maastricht University Medical Centre, Maastricht, the Netherlands; 2 Mondriaan Mental Health Trust, Department of Adult Psychiatry, Heerlen, the Netherlands; 3 King’s College London, King’s Health Partners, Department of Psychosis Studies, Institute of Psychiatry, London, United Kingdom; Hopitaux Universitaires de Geneve, SWITZERLAND

## Abstract

**Background:**

Routine Outcome Monitoring (ROM) should provide a dynamic, within-treatment forward feedback loop to guide individual treatment decisions across diagnostic categories. It has been suggested that the Experience Sampling Method (ESM), capturing the film of daily life adaptive processes, offers a flexible, personalised and transdiagnostic feedback system for monitoring and adapting treatment strategies. This is the first study that uses an ESM application (the PsyMate™) as a routine mobile-ROM (mROM) tool in an ambulatory mental health setting.

**Objective:**

To demonstrate adequate psychometric properties of the PsyMate™ app assessing both symptom severity levels as well as daily life functioning.

**Method:**

In a transdiagnostic sample of 64 outpatients, an mROM protocol (ESM for 6 days, at 10 semi-random moments a day) and a standard ROM instrument (HADS) were administered at baseline and at three-month follow-up. We measured positive affect (PA), negative affect (NA), quality of sleep, positive social interaction, activity-related stress, tiredness, and feeling unwell.

**Results:**

Subjects completed 53% of the measurements at baseline (*N* = 64) and 48% at follow-up (*N* = 29). Factor analysis and subsequent reliability analysis of PA and NA confirmed the two constructs. Significant and meaningful correlations were found between PA, NA and HADS scores (ranging from *r* = .4 to *r* = .7). Multilevel analyses yielded significant change scores for all measures.

**Conclusion:**

The ESM-based, transdiagnostic mROM tool can be used reliably in clinical settings: it shows adequate psychometric properties, as well as concurrent validity and sensitivity to change over time with respect to relevant ROM constructs. Person-tailored items can be added. In addition, mROM offers added value over standard symptom-based ROM, as it provides information on adaptive functioning in the daily environment of patients.

## Introduction

Routine Outcome Monitoring (ROM) in mental health care refers to the process of collecting clinical data, which can serve as feedback to guide informed treatment decisions [1]. ROM should be transdiagnostic, flexible and follow the flow of the clinical process. Furthermore, it must be adjusted to the needs and wishes of individual patients [2]. Given these requirements, there is considerable debate as to what degree clinical ROM measures in mental health care can serve as input to benchmark outcomes across clinicians and institutions–a process referred to as Benchmark-ROM [3]. Benchmark-ROM requires rigid standardization, both in terms of instruments and data collection, to allow cross-patient and cross-centre comparisons. The implementation of rigid benchmark-ROM negatively influences clinical ROM, which requires a more flexible approach [4–6]. Clinical ROM requires repeated assessments at specific time points over successive diagnostic and treatment phases. Although there is no agreed measure for ROM in mental health care, many ROM scales have been developed for use in clinical practice [7–9]. In the literature, ROM is often referred to as patient-reported outcome measures (PROM), stressing the need for unbiased assessments of outcome collected by the patient rather than the treating clinician [10, 11]. ROM instruments should be sensitive to change over time. Selected scales can be generic and, therefore, relevant across populations, or, in contrast, target specific patient groups. Moreover, ROM instruments should map several outcome domains, including psychopathology, daily life functioning, personal recovery, and quality of life [3, 12]. Unfortunately, current ROM implementations mainly focus on psychopathology [13], disregarding the other outcome domains. In addition, measures of psychopathology, expressed as total scores from traditional technical scales, do not reflect outcomes that are important to patients [14, 15]. Given that ROM is part of clinical routine, instruments should be time efficient. Furthermore, ROM should be set up in such a way that it allows the patient to become the empowered co-owner of the process of diagnosis and treatment. Therefore, ROM should be constructed as an informed forward feedback loop: the iterative process that includes clinical decision-making, outcome evaluation, treatment adjustment, and further outcome evaluation.

A wide variety of ROM instruments exists, often developed to assess psychopathology in specific patient populations (e.g., mood disorders and psychosis) using traditional rating scales that lack relevance to the daily life of patients. Comprehensive assessment batteries that incorporate all different outcome domains are cumbersome and time consuming. Inclusion of clinicians (or trained personnel) as assessors dramatically adds to the complexity and cost of data collection logistics and potentially introduces bias. In many cases, a trade-off has to be made between brevity and completeness [7]. Finally, ROM assessments often rely on retrospective assessments, which are vulnerable to recall bias and limit sensitive assessment of change [16]. Most individuals are poor assessors when asked retrospectively to assess emotional experiences [17]. The most essential problem with traditional ROM assessments is that they only marginally inform adequate treatment selection as they do not inform on personal goals in real life settings and are not set up in such a way that a constructive forward feedback loop arises. For customized treatment selection, symptom variability and contextual variation is relevant. Mental health problems and related disabilities occur in the flow of daily life. Effective treatment should address the issue that patients are most vulnerable and consider the coping strategies available within their daily routines. Traditional rating scales, completed in a clinical setting, are often not representative of daily life functional adaption. A successful recovery process assumes functional adaptation and increasing resilience against mental illness. Resilience factors, such as experience of positive mood states and the strength of social connections, are increasingly being recognized and used in mental health treatment [18].

Thus, ROM measurements should target contextual factors and positive mood states in addition to negative mood states and symptomatology, rather than being exclusively focused on negative symptomatic states. Experience sampling techniques take into account all of these factors. The *Experience Sampling Method* (ESM), also referred to as *Ecological Momentary Assessment* (EMA), is an umbrella term for several ambulatory assessment strategies that randomly sample mental state in the context, following the flow of daily life. ESM is a structured diary technique, in which patients are asked to complete short questionnaires in response to auditory cues (beep signals), at semi-random moments during the day, for a number of consecutive days. Patient assessments include momentary emotional experiences (e.g., positive and negative mood), symptoms, cognition, context information (e.g., activity, company and location), and an appraisal of the context. Because ESM is a repeated assessment strategy, additional constructs, such as stress-sensitivity or coping strategies, can be quantified [18]. ESM was proposed as a comprehensive, generic ROM instrument and has several advantages over typical ROM assessments [3, 18, 19]. First, ESM increases ecological validity, because reports are provided in the patient’s natural environment. Second, memory bias is minimized because patients report in the moment. Third, ESM is contextualized, allowing for a better understanding of the person-environment interactions that give rise to psychopathology. Being aware of environmental reactivity and pattern changes over time will aid the understanding of treatment progress [18, 20]. Fourth, ESM is highly relevant to the individual and is person-tailored when used as a clinical tool, thus creating transparency and facilitating patient engagement when discussing the data with the clinician. Last, ESM facilitates and enhances shared decision making processes, because patients are actively participating in the data collection, and data can be used to improve treatment efficiency. Study results show that ESM during treatment can enhance feelings of empowerment and the ability for self-management [21]. Across the psychopathology spectrum, ESM has been proven feasible and can be successfully applied, in combination with feedback, as a treatment method for depression [22]. Moreover, ESM has been found valuable for a range of uses, including ROM in clinical practice [23].

Van Os and colleagues studied the use of ESM as a mobile ROM (mROM) tool. They assumed that ESM data was sensitive to change over time, using several clinically relevant parameters. Observational analyses were performed using data from the treatment arm of a randomized control trial in patients diagnosed with major depressive disorder [24–26]. The results confirmed the assumption, with the greatest sensitivity to change found for measures of positive adjustment, positive affect, and increases of positive affect due to natural rewards in daily life. These results show that resilience factors, such as positive affect, are informative for outcome assessment. Positive mood states are relevant for treatment outcomes across mental disorders [27, 28] and can be seen as a generic trans-diagnostic indicator of underlying resilience that can be captured in ROM. Research showed that positive affect and negative affect are related but different dimensions, i.e., they are not the extremes of a single continuum. Positive affect is non-heritable and an indicator of resilience, whereas negative affect is highly heritable and an indicator of vulnerability [29]. As both dimensions are important in clinical context, both should be captured in ROM. With the advent of modern mHealth applications for use on mobile devices, such as smartphones, ESM can now be carried out cheaply and routinely [23]. We refer to mHealth ESM for the purpose of ROM as *mROM*. There are multiple ESM mobile applications available (e.g. https://www.lifedatacorp.com/, http://experiencesampler.com/, https://pielsurvey.org/). Here, we present the first study that uses a mobile device to implement mROM in an ambulatory mental health setting, using an open trial design with PsyMate™, one of these ESM mobile applications (www.psymate.eu).

### Objective

The aims of this study were twofold. First, to demonstrate transdiagnostically adequate psychometric properties of routine mROM with a PsyMate™ moderated mROM application and second, to investigate sensitivity to change during treatment. Based on previous work, it was expected that (i) the psychometric properties of the PsyMate™ method would be adequate and suitable for therapeutic monitoring; and (ii) change over time could be demonstrated for a series of variables (e.g., mood, activity-related stress, and sleep quality) indexing both psychopathology and daily life functioning. In this regard, we expected to find a weak to moderate association between a traditional rating scale (the Hospital Anxiety and Depression Scale [30]) and the mROM mood measures, leaving sufficient room for the added value of mROM.

## Methods

### Sample

Patients were recruited at the outpatient mental health service of the Maastricht University Medical Centre (MUMC+). In total, 115 consecutively attending patients were asked to participate in routine mROM, of whom 75 provided their consent. All patients were 18 years or older and capacity for consent was established by the psychiatrist providing the care to the patient. Exclusion criteria for inclusion in the analysis were not being able to read Dutch or not being able to handle a mobile device with the PsyMate™ app.

### Procedures

#### Analysis design

mROM with the PsyMate™ app is applied routinely in MUMC+. There were two measurement periods, one at baseline (t = 0), the second at follow-up approximately three months later (t = 1). Due to logistical reasons, the second visit did not always take place exactly 3 months later (*M* = 111.6 days later, *SD* = 27.3, range 80–189 days). Each measurement period consisted of six consecutive ESM sampling days and the administration of a traditional ROM questionnaire at the beginning of the ESM sampling days.

#### Briefing

During the baseline session, patients were helped while downloading the Psymate™ application on their smartphone via the App Store or Google play store. A specific code was required to enter the study protocol. However, a free demo app is available in different languages, for those wishing to implement the app in routine clinical practice. In case a patient could not use his own device, an iPod was provided for the duration of the mROM period. A clinician explained the PsyMate™ procedure during a briefing session and coached patients through the PsyMate™ items. Patients were instructed to continue their normal routine during the Psymate™ data collection period. After the briefing, a sampling period of six consecutive ESM days started, not including the briefing day on which the PsyMate™ protocol was activated.

The standing medical ethical committee approved the anonymous use of routine clinical data, if patients provided informed consent. Thus, all patients provided informed consent to use routine clinical data for the purpose of scientific investigation.

#### mROM

For the purpose of mROM, the Psymate™ application was programmed to emit 10 random beeps each day. Signals notify when a short questionnaire has to be completed (lasting approximately 1 minute). Beeps were semi-randomised in ten blocks of 90 minutes, between 7:30 AM and 10:30 PM. The questionnaire consisted of 13 mood items, 5 context items (what, where, and with whom the patient was spending time and whether they were enjoying it), 5 items about important events happening since the last beep, 1 item questioning specific somatic complaints, 5 items assessing patients’ physical condition, and 1 item assessing levels of beep disturbance. In addition to the beep questionnaire, patients filled in some extra questions at the beginning and the end of each day. The morning questions were related to the quality of sleep of the night before, while the questions in the evening required the patient to give an estimate of their average mood and somatic complaints over the past day. Most items were presented on a 7-point Likert scale, ranging from 1 (not at all) to 7 (very).

In order to assess change over time, items were clustered to form measurable constructs. Previous work showed positive and negative mood items reliably form a Positive Affect (PA) and a Negative Affect (NA) cluster [31]. In this analysis, PA consisted of the items: I feel cheerful, satisfied, relaxed and globally feeling well. The remaining nine mood items (I feel lonely, guilty, worried, down, threatened, insecure, irritated, frightened, and suspicious) formed the NA scale. The quality of sleep was assessed using the (ordinal-coded) items: (1) time needed to fall asleep, (2) number of times the person woke up during the night, (3) the time lying awake before getting up, (4) whether the person felt rested, and (5) how the person globally felt about their sleep quality last night.

Approximately three months after the initial visit, patients were asked to participate again in the second part of the PsyMate™ mROM procedure.

#### Debriefing

After each sampling period, a debriefing session was scheduled. Patients were asked whether the past week was representative for their daily life and whether the Psymate™ interfered with their thoughts, feelings, activities, or social contacts. Furthermore, patients were asked whether unusual incidents occurred, and to what degree the use of Psymate™ was bothersome.

#### HADS

The Hospital Anxiety and Depression Scale (HADS) [30] is a reliable and validated rating scale [32, 33] which is often used for mental health ROM purposes worldwide, particularly in somatic hospital settings [34]. The HADS consists of fourteen items (scaled from 0 to 3) assessing aspects of anxiety and depression experienced during the last week. The HADS was administered twice digitally (via the Psymate™) at the end of each briefing session.

### Statistical analyses

Analyses were performed to investigate the structure of the 13 mood items. First, a principal component analysis (PCA) was conducted on these items with orthogonal rotation (varimax), requiring two factors to be extracted. Second, a reliability analysis (i.e. Cronbach’s alpha) was performed on each factor to determine the internal consistency of each scale.

The data collected with ESM have a multilevel structure—successive beeps (level 1) are nested within patients (level 2). Multilevel regression analyses took the variability of both levels into account. With respect to the analyses of change, there were 7 *a priori* dependent variables: NA, PA, quality of sleep, preferring to do something else (activity-related stress), enjoying company (social stress), feeling unwell, and feeling tired. The following items were incorporated as covariates in the multilevel models: age, sex, a dichotomous variable indicating whether a patient participated in one or both measurement periods, and DSM-IV-TR diagnosis prior to participation (i.e., a dichotomous variable indicating whether or not a patient had been previously diagnosed with a mental disorder (e.g., depressive disorder, panic disorder, somatoform disorder, no diagnosis, etc.)) (35). Additionally, when examining the variable *feeling unwell*, a dichotomous variable describing the presence or absence of somatic complaints was taken into account and added as a covariate in the statistical model. The predictor variable of main interest was measurement period, indicating a possible change over time.

Analyses were carried out using SPSS Statistics version 23.0. Two-sided p-values < .05 were considered significant. All dependent variables were checked for approximately normal distribution before performing further analyses.

## Results

### Sample

In the period from February 2015 until May 2016, 75 patients at the MUMC outpatient mental health service fulfilled criteria for inclusion in the analysis. In case a patient completed less than ten beeps during a measurement period (either baseline or follow-up), the measurement period was excluded from further analysis. There were 11 patients who did not complete sufficient beeps during both measurement periods, leading to a final dataset of 64 patients. Thirty-six patients completed one measurement period, 28 patients completed both measurement periods. A logistic regression model examined whether these two groups differed with respect to age, sex and being diagnosed (yes or no). This was not the case for either the omnibus model (-2 *LL* = 78.9; *df* = 3; *p* = .18) or for the univariate tests (all *p*-values > .075).

The average age of the *N* = 64 analysable group was 48.7 years (*SD* = 13.9, range 18–73). There were 39 women and 25 men. All but 11 patients received a clinical diagnosis by the treating psychiatrist, based on DSM-IV-TR criteria. The 11 non-diagnosed patients did experience a degree of mental discomfort and were therefore included in the analysis. Diagnoses included depression (*N* = 25), panic disorder (*N* = 18), somatoform disorder (*N* = 4), posttraumatic stress disorder (*N* = 2), bipolar disorder (*N* = 1), anxiety disorder (*N* = 1), dysthymia (*N* = 1) and psychotic disorder (*N* = 1).

### App statistics

The complete protocol presented 120 beeps per patient (6 days x 10 beeps x 2 measurement periods). At baseline, the response percentage was 52.7% of 3780 presented beeps, comparable to the 48.2% of 1740 presented beeps at follow-up.

In October 2015, an update of the PsyMate™ app was introduced. Since then, the number of errors concerning the Internet connection considerably reduced. The proportion of valid beeps with no missing items within the beep increased substantially after this update: from 71.1% to 86.2%.

### Compliance

**Fig 1:**
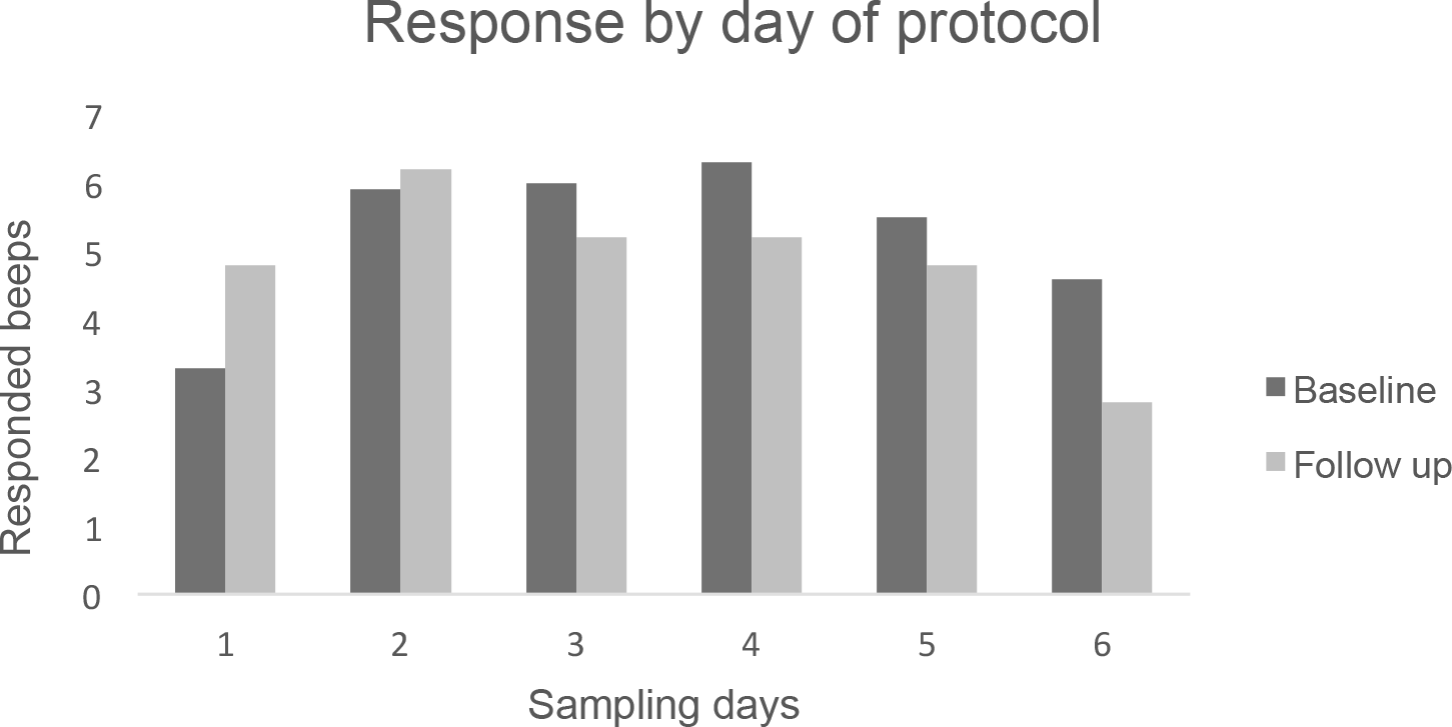
This figure shows the average number of beeps that were completed per day per subject, both for the baseline assessment and the three-month follow-up assessment, over the 6 day ESM period.

**Fig 2:**
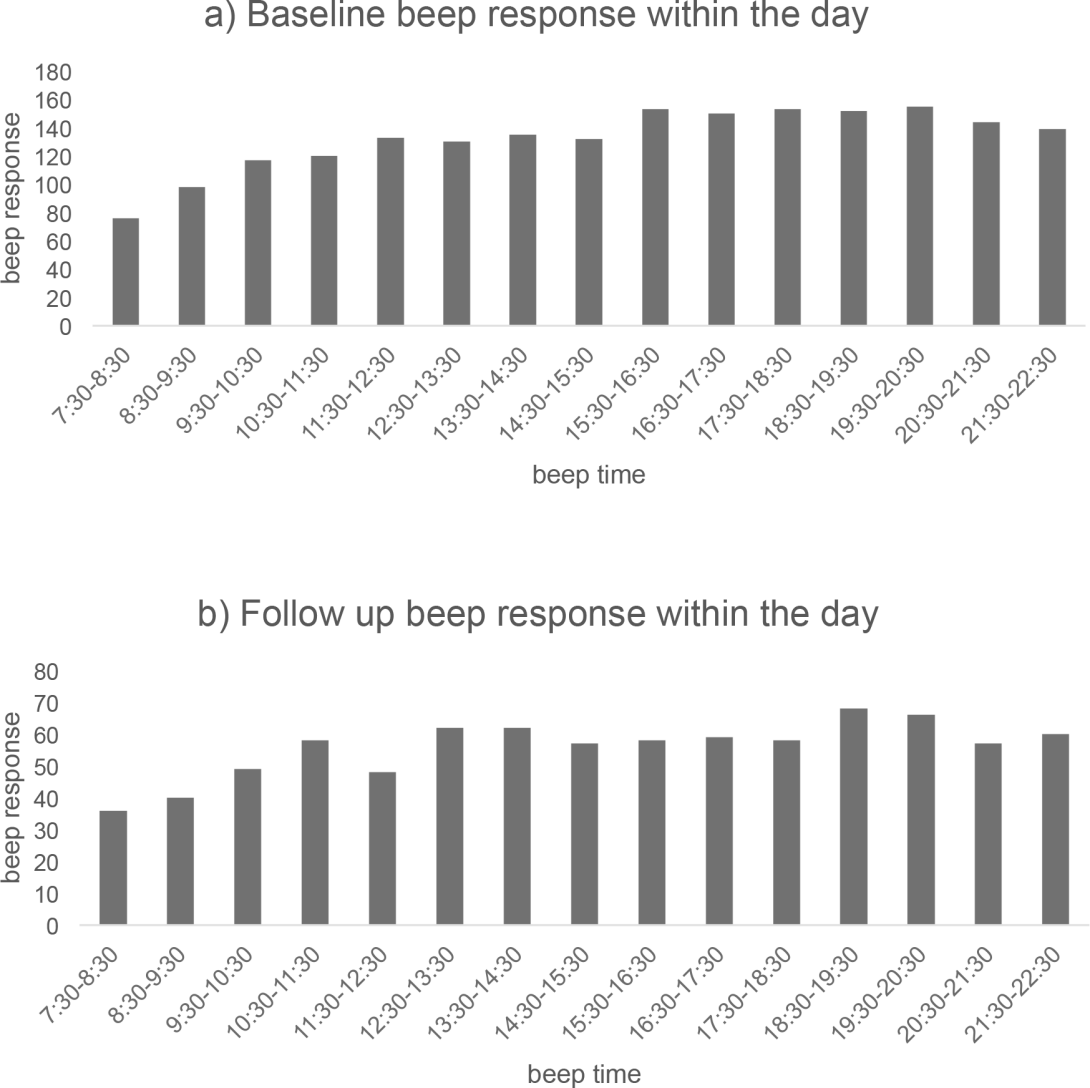
This figure shows the average number of responded beeps, per time window, within a day. (A) Number of beep responses within the day, derived from baseline ESM data. (B) Number of beep responses within the day, derived from the three-month follow-up ESM data.

There was no suggestion of relevant differences in response rates between the days of the week at either baseline or three-month follow-up. However, the data suggest that there is a difference in response rate over consecutive days of the ROM protocol (Fig 1). During the first day and last day of the protocol, patients tend to complete less beeps than on the remaining days. At both baseline and follow-up, a reduction in response rate over time was apparent. At follow-up, the reduction started two days earlier (day 3), compared to baseline. Regarding the hours of the day, there was a slight increase in response over time, as illustrated in Fig 2. The lowest response was in the early hours of the day (7:30 am–10:30 am).

### Factor structure of PA and NA

The principal component analysis of the 13 PA and NA items resulted in two factors, each having a value larger than 1 (7.1 and 1.5 respectively), with a cumulative percentage of explained variance of 66.1%. An item was assigned to a factor when the factor loading exceeded .45 with a difference larger than .1 for both factor loadings. Using these criteria, all items could be assigned unambiguously to either the PA or the NA factor. As a next step, a reliability analysis was performed. Cronbach’s alphas for factor 1 and 2 (NA and PA) were .91 and .92 respectively.

Consequently, the PA subscale was constructed by computing the sum of the 4 positive affect items, the NA scale by computing the sum of the 9 negative affect items. Sub-scores containing missing items were computed by inserting the mean value of the available items. A maximum of two missing items for PA and 4 items for NA was allowed. PA and NA could not be computed for 41 beeps, leaving an analysable number of beeps (records) of 2832.

### Validation analyses

A correlation matrix with the patient-level means of the PA and NA scores (beep-level data) and the patient-level means of HADS total, HADS anxiety, and HADS depression was computed (Table 1). The results confirmed the a priori hypothesized relationships between these five variables. Next, an analysis was performed to investigate whether a change in PA and NA over time correlated with a change on the HADS total score. Significant correlations between change scores (baseline versus 3 months) were found: ΔPA with ΔHADS -0.634 p = 0.002, ΔNA with ΔHADS 0.603 p = 0.004.

**Table 1:** Pearson correlates between positive affect scores, negative affect scores and hamilton anxiety and depression scale scores.

Variables	1	2	3	4	5
1. NA mean	-				
2. PA mean	-.71***	-			
3. HADS anxiety	.71***	-.51***	-		
4. HADS depression	.56***	-.45***	.66***	-	
5. HADS total	.66***	-.51***	.89***	.92***	-

Legend. Correlations between 1 and 2 were performed over *N* = 63. All others were performed over *N* = 59.

NA = Negative affect, PA = Positive affect, HADS = Hamilton Anxiety and Depression Scale.

NA and PA represent mean scores over all subjects for one measurement period.

HADS anxiety, HADS depression, and HADS total represent subject scores for one measurement period.

*p < .05.

**p < .01.

***p < .001.

### Sensitivity to change

Analyses were performed with multilevel regression to examine whether the positive change that was *a priori* expected could be demonstrated (Table 2). Each model used the same set of predictor variables (i.e., a dichotomous variable for the assessment moment, and age, sex, being diagnosed yes or no, and participation in both measurement periods as covariates). As reported in Table 2, the mROM Psymate™ was able to detect significant changes over time in all the variables.

**Table 2:** Multilevel regression model estimates for the effects of measurement period on several variables.

Dependent Variables	B (*SE)*	t-values	p-values
Positive affect	.65 (.19)	3.45	.002
Negative affect	-.37 (.14)	-2.61	.015
Quality of sleep	1.89 (.85)	2.23	.033
Positive social interaction	.44 (.15)	3.69	.001
Activity-related stress	-.37 (.18)	-2.10	.046
Feeling tired	-.54 (.18)	-2.94	.007
Feeling unwell	-.67 (.21)	-3.17	.003

Legend. The analyses are based on 2874 beeps nested within 64 persons. SE = standard error.

## Discussion

This study examined the applicability of a routine mobile-ROM tool within an ambulatory mental health setting. The PsyMate™ application was used to implement experience sampling methodology (ESM). The first aim was to demonstrate adequate psychometric properties of mROM, by investigating the reliability and validity of the mROM application in PsyMate™. The second aim was to investigate sensitivity to treatment change using PsyMate™ data.

Results demonstrated adequate psychometric properties of the PsyMate™ app when used with the present mROM protocol. The reliability of the method was examined by exploring user characteristics of the PsyMate™. Compliance rates during both the baseline and follow-up periods (a period consisted of 60 possible beeps per person; 10 per day, for 6 days), were around 50 percent, in terms of beeps completed. Although ESM compliance may seem lower compared to previous ESM findings, which showed compliance rates around 80 percent [35], sufficient beeps were completed for reliable data analyses. There are a number of possible reasons for the reduced compliance. Even conscientious patients miss some beeps due to daily life demands. Furthermore, the compliance rates found in earlier studies were based on traditional sampling techniques, using booklets combined with wristwatches or Personal Digital Assistants. Therefore, a direct comparison to the PsyMate™ app is misleading. In addition, there were technical issues with the first release of the app, leading to data loss when the Wi-Fi communication was unstable. After a system update, these problems were reduced. Hence, the compliance with the PsyMate™ app is considered acceptable. Different strategies may be used to improve compliance. The briefing session is important. It should create transparency about the reasons to collect mROM data: to optimize treatment, participate in clinical research and/or to comply with administrative requirements. The briefing session is the start of building an alliance in which clinicians try to understand needs arising in the context of daily life. It helps to discuss ESM sampling situations that shed light on aspects of resilience and vulnerability (with the possibility to customize ESM-questionnaires if necessary). Elements of ‘gamification’ can be included to motivate patients to fill in the ESM sampling sheets. A crucial factor is the personalized feedback on daily life strengths and vulnerabilities. The PsyMate™ includes an automated web-based ESM feedback that clinicians and patients can consult. The option should be better advertised because it makes data collection more relevant to individual patients. Looking at the response behaviour at week level, few differences were found across the days of the week, although the first and last day of the ESM protocol were characterised by slightly fewer responses. During the baseline assessment, the first day showed the lowest response rate, whereas in the follow-up period, the last day had the lowest response rate. A lack of familiarity with the ESM routine is arguably the reason for the low response rate on the first day, while the anticipation of completing the ESM period might account for the low numbers on the final day [36]. Considering data at day level, fewer beeps were completed in the morning hours. Again, this is to be expected, since patients were instructed to follow their own daily pattern and sometimes were still asleep at the early pre-programmed moments. When considering attrition rates, only 28 patients completed both measurement periods, or 37% of the 75 patients initially included. Some patients were excluded because there was not enough data and others did not complete the follow-up measurement. The loss of patients at follow-up could indicate that the method is too burdensome or not experienced as relevant. This seems unlikely, given that previous research has shown that the method was feasible in patients with a wide variety of mental disorders [23] and current findings indicate feasibility within a single measurement period. Furthermore, a substantial proportion was already discharged from the mental health service by the time of the three month follow-up, as can be expected within the dynamics of a hospital outpatient mental health setting. After three months, patients that were seen in the context a diagnostic assessment would already have been referred back. Other patients would have discontinued treatment in the high-attrition risk context of referral from a somatic department to a mental health setting. To a degree, limited follow-up may reflect the natural flow of a general hospital mental health care setting. Future research should further examine this issue. From a clinical perspective, mROM is most relevant at the beginning and during treatment, when data can be used to customize interventions. Nearer the end of treatment, patients motivation may naturally decrease as they start to leave the episode of mental distress behind [37].

The constructs Positive Affect (PA) and Negative Affect (NA) were selected based on the existing ESM literature [31]. Factor analysis of these items confirmed the structure of the two concepts and the subsequent reliability analyses yielded excellent internal consistency coefficients. To assess the concurrent validity of mROM, comparisons where made between the traditional ROM questionnaire used in the hospital (HADS; assessing anxiety and depression) and the PsyMate™ moderated mROM protocol. Overall, significant and clinically relevant correlations were found between the PA and NA constructs on the one hand, and the HADS (total, anxiety, and depression) scores on the other. Although the measures show substantial overlap, the ESM based PsyMate™ moderated mROM protocol offers a unique contribution to clinical routine outcome assessment given that recall bias and contextual biases are controlled for to a great extent. In addition, the ecological measures within the mROM protocol inform on daily life adaptive functioning. Seven measures (PA, NA, positive social interactions, quality of sleep, activity-related stress, tiredness, and feeling unwell), considered relevant in mental health care, were assessed and compared over baseline and follow-up. All measures changed significantly over time. Thus, the mROM measurements are sensitive to change over time. At follow-up, scores on NA, activity-related stress, tiredness and feeling unwell were reduced, whereas scores on PA, positive social interactions, and quality of sleep were increased. This overall sensitivity to change is in line with previous ESM research [19, 21].

The study sample was heterogeneous, although mood and anxiety disorders were most prevalent in this general hospital setting. Despite a degree of heterogeneity, the same Psymate™-moderated mROM protocol was used transdiagnostically for all patients. The ESM questionnaires used in different mental health target populations typically share 80% of the items, in order to generically assess contextualized symptom variability and well-being in daily life [20]. The mROM tool shows initial usefulness as a transdiagnostic instrument. However, the small sample size and limited heterogeneity in our sample warrant further investigation across more diagnostic groups. Moreover, in contrast to traditional ROM tools, the data collection is not restricted to symptom intensity, but also includes assessment of functioning and quality of life. The ESM procedure allows for patient-reported outcome measures that index adaptive daily life functioning, avoiding potentially biased data collection methods based on clinical interviews conducted by the treating therapist. Self-reports of mental states can have their own biases; the ESM sampling procedure is designed to avoid these risks, which may be especially advantageous in general hospital patients with somatic complaints due to underlying mental conditions [38, 39].

**Fig 3:**
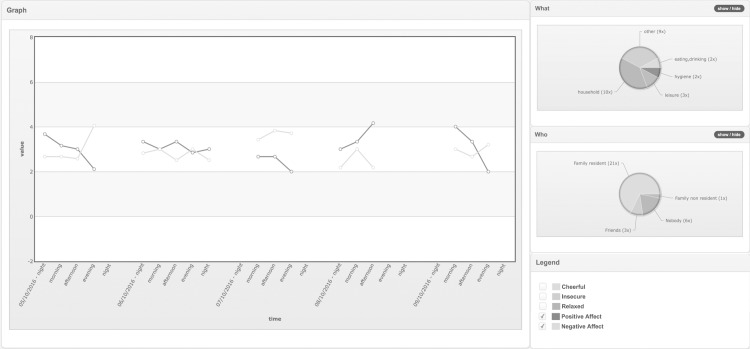
This figure is an example of the PsyMate™ ESM feedback, provided on a dedicated website with choice of ‘contextual’ or ‘functional’ analysis. The data (here five consecutive days) are displayed over time (here clustered in dayparts; morning, afternoon …). Aggregated Likert scores of the scale items (here positive- and negative affect is selected) reflect the mental state at the time points. What and Who pie charts display the time budgets for the sampling period. In an interactive feedback meeting with the patient, the clinician can select options of the pie chart to break down the subject’s responses and assess whether mental states are contextualized (subject feels better in one situation, compared to the other) or zoom in on specific moments, that reflect vulnerability (crisis) or resilience (coping).

The logistics of ESM data sampling, and thus the mROM data-collection, were simplified by using modern smartphones. Patients can use their own device to collect data in daily life. The Psymate™ methodology allows customization; beeps can be programmed, questionnaires adapted and person-tailored items can be added. Collected data is immediately sent to the database whenever a connection is available and remains continually available for feedback through a website with understandable graphs and figures (Fig 3). The improved logistics broaden the applicability of the ESM method from limited use in research centres to routine use in clinical care [40].

ROM is often positioned as a management tool, which suggests that ROM-data can be used to benchmark different centres. ESM methodology, applied as an mROM tool, assures that ROM data has a far broader applicability. The high level of patient involvement increases its clinical relevance to users of services [41]. Moreover, clinical and patient relevance is further impacted by the possibility of accessing and viewing the data independent from services. Thus, in order to make ROM relevant to both the patient and the clinician, ESM may be rolled out routinely.

## Supporting information

S1 Data mROM data for multilevel data analyses. (XLSX)
